# Femtosecond Laser Deposition of Germanium Selenide onto Silicon Platform at Different Substrate Temperatures

**DOI:** 10.3390/nano12122003

**Published:** 2022-06-10

**Authors:** Kheir S. Albarkaty, Eric Kumi-Barimah, Jian Zhang, Zhiyong Yang, Gin Jose

**Affiliations:** 1 Department of Physics, Umm Al-Qura University, Makkah 24382, Saudi Arabia; ksbarakati@uqu.edu.sa; 2 School of Chemical and Process Engineering, University of Leeds, Leeds LS2 9JT, UK; g.jose@leeds.ac.uk; 3 Shanghai Institute of Ceramics, Chinese Academy of Sciences, Shanghai 200050, China; jianzhang@mail.sic.ac.cn; 4 School of Physics and Electronic Engineering, Jiangsu Normal University, Xuzhou 221116, China; yangzhiyong@jsnu.edu.cn

**Keywords:** femtosecond pulsed-laser deposition, chalcogenide glass, germanium selenide (GeSe_4_)

## Abstract

Germanium selenide (GeSe) thin films were fabricated by employing femtosecond pulsed-laser deposition (fs-PLD) on silicon (100) substrates at various substrate temperatures, ranging from 25 °C to 600 °C. The thin films’ surface morphology qualities and optical properties were studied by utilising transmission electron microscopy (TEM) and X-ray diffraction (XRD). The X-ray diffraction result signifies that the thin films deposited on the silicon at a substrate temperature below 400 °C were amorphous Ge-Se. In contrast, those grown at 400 °C and above exhibited crystallised peaks of Ge-Se orthorhombic and tetragonal structures. The deposition growth rate of the thin films was also found to decrease substantially with increasing substrate temperature. These results show that the fs-PLD process has great potential for fabricating good quality Ge-Se thin film. This technique could enable the manufacture of modern optoelectronic devices for applications in optical communication, sensing, and ovonic threshold switching for the high-density crossbar memory array.

## 1. Introduction

For the past six decades, silicon photonics has attracted broad interest in research into the development of optoelectronic devices aimed at optical communication, sensing, and optical or electrical data storage applications [1,2,3,4]. However, silicon has robust nonlinear absorption and free-carrier effects, limiting the nonlinear response speed needed to process signals with a high data rate [4,5,6]. Therefore, it is essential to investigate new materials to counter some of silicon’s shortcomings. Chalcogenide glasses are desirable materials for developing active and passive devices due to their remarkable optical and electrical properties, such as low-energy phonons [7,8], photo-induced phenomena [9,10], wide-transparency windows [11,12], and high nonlinear and linear refractive indices [13,14]. These excellent characteristics of chalcogenide glasses make them good candidates for developing the next generation of on-chip photonic platforms for ultrafast all-optical signal processing or optical switching (memory and selector units); for developing a single-mode planar waveguide for optical communication; and for broadband supercontinuum generation in mid-IR for spectroscopy and sensing [15,16,17,18].

Germanium selenide (Ge-Se) semiconductors belong to the chalcogenide glasses which contain elements, found in Group IV and VI of the periodic table. Both amorphous and crystalline Ge-Se compounds have become very important in material science and engineering, because of their excellent technological applications in infrared optics [19,20]. Understanding the fundamental properties of these novel materials, in both bulk and thin film, would establish a relationship between electron physics and potential device applications. The ovonic memory-switching behaviour of Ge-Se materials depends mainly on the phase transformation from amorphous to crystalline, but it can remain in the amorphous state without a phase transition. For instance, Sulitanu et al. [15] investigated amorphous Ge_46_Se_54_ thin films fabricated by employing thermal vaporisation in a vacuum to evaluate their potential for switching phenomena. It was observed that switching could be initiated from those Ge-Se thin films tested. which is promising for switching devices in the optoelectronic circuit. In addition, such amorphous Ge-Se thin films are of interest for applications in manufacturing antireflection coatings, filters, and other optical devices capable of transmitting infrared light [16,17,18]. Furthermore, most chalcogenide semiconductor thin films are sensitive to photo-induced exposure, which produces a phase transition of their structures from amorphous to crystalline. The photo-induced effects of the transition from amorphous bulk glass to crystalline also include changed optical properties such as bandgap energy and refractive index. Several varieties of chalcogenide glasses, such as amorphous selenium, and the stoichiometric IV-VI mixtures GeSe_2_, GeS_2_, and SiSe_2_, with this effect, have been reported [21,22,23].

Recently, Ge-Se thin films have been produced using various fabrication methods, including thermal vacuum evaporation [24,25,26], chemical vapour deposition (CVD) [27], pulsed-laser deposition (PLD) [28,29,30], and others. Among these techniques, the fs-PLD method has been recently employed to fabricate many chalcogenides and rare-earth-doped chalcogenide glass thin films [31,32,33,34]. The ultra-fast fs-PLD process has several advantages including simple-to-use fabrication of a uniform thin film from target materials without altering the stoichiometric composition, the absence of any post-annealing requirement, and easy control of the deposition parameters such as substrate temperature, substrate–target distance, chamber pressure, and laser fluence, all of which optimise the process [30].

This work aimed to deposit or dope Ge-Se on/into silicon substrates at various substrate temperatures using fs-PLD. Different characterisation methods were employed to investigate the effects of temperature upon surface morphology, microstructure, composition, and optical properties of as-deposited Ge-Se thin films.

## 2. Experimental Section

### 2.1. Fabrication of GeSe_4_ Glass Target and Thin Film

The GeSe_4_ glass was synthesised using the conventional melt-quenching technique [35,36]. Germanium lumps of 5N purity and selenium particles of 6N purity were weighed and loaded into a low-OH silica tube in a glove box filled with dry nitrogen. The tube containing the elemental materials was then connected to a vacuum system. Once the pressure was below 7.53 × 10^−6^ Torr, the tube was sealed using an H_2_-O_2_ flame. After that, the tube was put into a rocking furnace and heated to 850 °C. After being homogenised at this temperature for 12 h, the mixture in the tube was quenched in water. The formed glass was finally annealed at 160 °C for 3 h.

A femtosecond laser (Ti: sapphire) with a central wavelength of 800 nm, a 1 kHz repetition rate and 100 fs pulse duration was used to grow Ge-Se thin films. The silicon substrates were cleaned thoroughly, first with deionised water at a temperature of 50 °C, then with acetone and isopropanol. The cleaned substrates and high-purity amorphous Ge-Se target material were mounted on separate holders in a stainless steel vacuum chamber. The rotation speeds of the holders of the target material and substrate were set to a constant 40 rpm and 20 rpm, respectively, throughout the film fabrication process. This was to ensure that the target material maintained a constant uniform surface, and thus preventing any disorientation of the plasma plume towards the substrate over time. The distance between the substrate and the GeSe_4_ target was set at 70 mm. The vacuum chamber was initially pumped down to a base pressure of about 10^−6^ Torr to eliminate any water residues within the chamber and then kept under this pressure throughout the entire deposition process. The substrate temperatures of the samples fabricated were 25 °C, 200 °C, 400 °C, and 600 °C, respectively, with a deposition period of 4 h for each sample. The thin films were prepared with an fs-laser energy level of 40 µJ, which was focussed onto the amorphous Ge-Se target at an angle of 60° to create a plasma plume directed perpendicular to the substrate surface. At the end of each fabrication process, the substrate was allowed to cool down to room temperature before the sample was removed from the chamber.

### 2.2. Characterization of Thin Film

The surface morphology, film quality, and thickness, as well as the elemental composition of the thin films prepared, were characterised using scanning electron microscopy and FEI Helios G4 CX DualBeam focused ion beam scanning electron microscopy (FIB-SEM), transmission electron microscopy (TEM and FEI Titan Themis Cubed 300 TEM (transmission electron microscopy)), and high-resolution monochromated FEG-SEM (field emission gun scanning electron microscopy). The structural properties of as-deposited Ge-Se films were studied by utilising X-ray diffraction (XRD). Philips X’Pert measured the XRD patterns of the films with Cu Kα radiation (λ = 1.54056 Å) at 40 kV and 100 mA. Each sample was scanned for 60 min using a diffractometer angle ranging between 10° and 60° with a step size of 0.033°. The Raman spectrum of the GeSe_4_ thin films was measured at room temperature using a Raman spectrometer (Renishaw via Raman microscope) at a wavelength of 514.5 nm from an Ar-ion laser operated at 20 mW. Room-temperature transmittance and reflectance were measured with a Perkin Elmer Lambda 905 UV–VIS–NIR Spectrophotometer equipped with an integrating sphere in the wavelength range of 250–2000 nm to determine the optical bandgap of the fabricated thin films.

## 3. Results and Discussion

**Figure 1:**
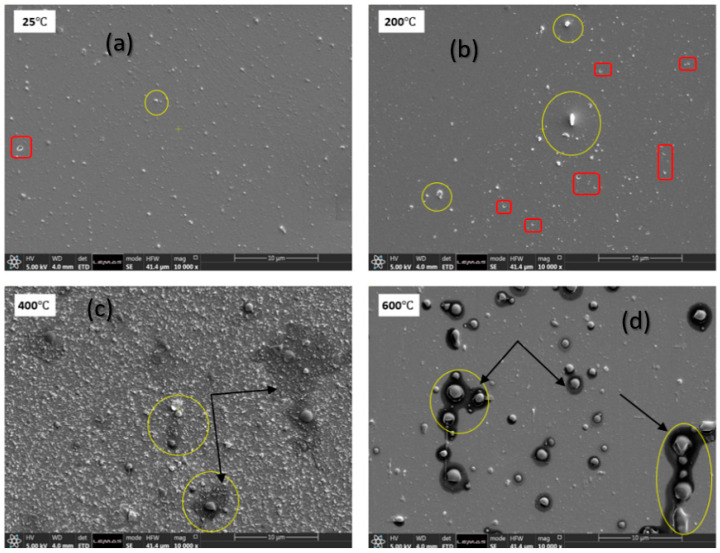
Top surface backscattered SEM mages of Ge-Se thin films deposited on a silicon substrate at various temperatures: (**a**) 25 °C, (**b**) 200 °C, (**c**) 400 °C, and (**d**) 600 °C.

Figure 1 shows the SEM images of the GeSe_4_ thin film deposited on the silicon substrate at various temperatures. The sample fabricated at 25 °C exhibits small, scattered droplets of particles in isolated places on the surface of the substrate, which are circled in yellow. In addition, the surface morphology of the film demonstrates a homogeneous and smooth surface without any agglomeration. Similarly, as the substrate temperature increases from 25 °C to 200 °C, the droplets of nanoparticles (circled in yellow and red) distributed on the surface of the silicon substrate remain the same as the sample fabricated at 25 °C, thus indicating that the thin films are very compact. However, as the temperature is elevated to 400 °C (Figure 1c), there is an increase in the surface porosity and roughness of the film. Finally, the sample deposited at the highest temperature of 600 °C reveals uneven large particles or droplets on the substrate surface (circled in Figure 1d). The changes in the surface morphology of the thin films as the substrate temperature increases is attributed to the glass transition temperature of GeSe_4_ glass occurring at 160 °C. Therefore, as the substrate temperature increases above 300 °C, the result in lower viscosity and increased volatility, which could be linked to structural and chemical transformations of the deposited layer, or by Ge-Se diffusing into the silicon substrate.

**Figure 2:**
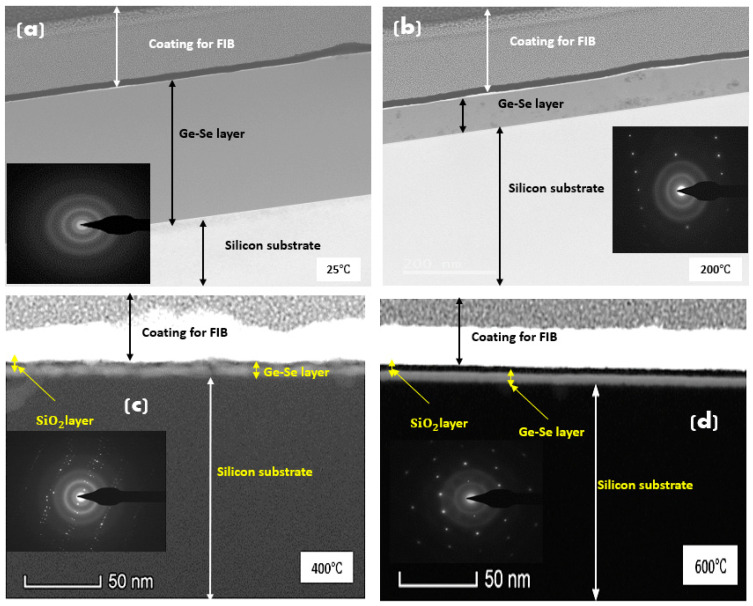
TEM cross-section images of Ge-Se layers formed on silicon substrates by fs-PLD at various substrate temperatures (**a**) 25 °C (S25), (**b**) 200 °C (S200), (**c**) 400 °C (S400), and (**d**) 600 °C (S600).

Cross-sections of the Ge-Se thin films deposited on/onto silicon were obtained via focused ion beam (FIB) lithography and examined on the transmission electron microscope (TEM). Figure 2a–d show TEM images of the cross-sections and selective area electron diffraction pattern (SAED) patterns of the investigated Ge-Se thin films obtained using FIB lithography. Figure 2a shows a closer view of the TEM image of the thin film deposited at room temperature (25 °C), which displays a uniform cross-section of the Ge-Se layer sandwiched between the coated layer on top and pure silicon below. The cross-section of Ge-Se thin film looks darker than the silicon layer as Ge and Se are heavier elements than silicon. It is important to mention that the deposited layer is smooth without nanoparticle clusters or defects. Nevertheless, as the substrate temperature increases from 25 °C to 200 °C, the TEM image (Figure 2b) shows occasional defects within the Ge-Se thin film layer. These defects may be composed of a different phase of Ge-Se. The TEM cross-sections of samples deposited at 400 °C and 600 °C indicate that higher substrate temperatures have significant effects on film thickness and quality, as shown in Figure 2c,d. The plain view of TEM images reveals an average Ge-Se thin film thickness of ~400 nm for sample S25, ~150 nm for sample S200, and ~7 nm for samples S400 and S600. This finding indicates that the deposited material is volatile at higher temperatures and almost completely disappears from the surface of the silicon substrate at 600 °C.

The SAED pattern of sample S25 confirms the presence of amorphous material owing to the existence of spherical overlap rings around the central spot., while the remaining samples S200, S400, and S600 exhibit a mixture of amorphous and polycrystalline features because of interdiffusion between the silicon substrate and GeSe_4_, or diffusion of the GeSe_4_ into the silicon substrate.

**Figure 3:**
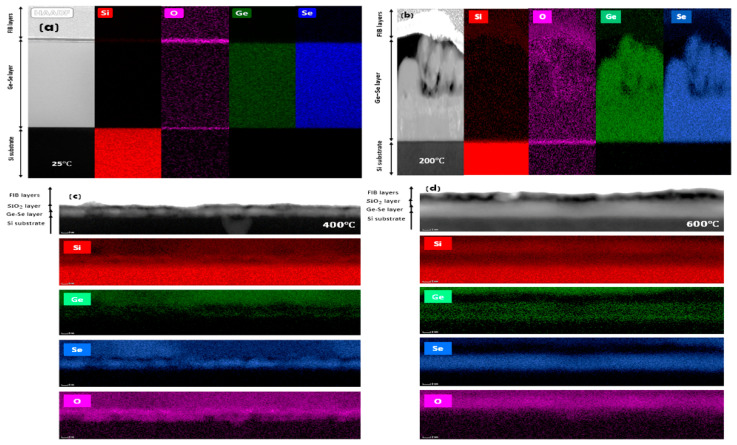
EDX-STEM elemental map images of Ge-Se thin films prepared on silicon at (**a**) 25 °C, (**b**) 200 °C, (**c**) 400 °C and (**d**) 600 °C.

Energy-dispersive X-ray (EDX) analysis of the four TEM cross-sections of the thin films fabricated at various substrate temperatures was performed in scanning-TEM (STEM) mode on the TEM to understand the elemental distribution within the thin film layer. The elemental EDX-STEM maps of silicon (Si), germanium (Ge), selenium (Se), and oxygen (O) of the samples prepared at 25 °C and 200 °C are shown in Figure 3a,b without any intermixing of the deposited Ge-Se thin film, the silicon substrate, and the thin silica layer on it. This shows that Ge, Se, and O elements are uniformly distributed within the Ge-Se thin film. Elemental EDX-STEM maps of Ge, Se, and O from the sample prepared at 400 °C are shown in Figure 3c. The deposited Ge-Se layer exhibits slight intermixing and interdiffusion behaviour with the silicon substrate at the interface. For the 600 °C samples, as shown in Figure 3d, the EDX-STEM maps show that the deposited Ge-Se layer has no evidence of intermixing. Instead, GeSe_4_ is able to diffuse into the silicon to form a thin film layer within the silicon substrate. This diffusion of GeS_4_ into a silicon substrate at an elevated temperature can be described as thermally-induced and assisted by the thin silica layer present on the silicon.

Additionally, it was evident that the Ge and Se distribution varied across the thin film at higher deposition temperatures (400 and 600 °C), as shown in Figure 3c,d. However, at the highest substrate temperature (600 °C), the Se concentration dominates in the thin film layer via diffusion, when compared to Ge. Thus, the fs-laser–target material interaction induces rapid evaporation of Ge at higher substrate temperatures. This depends on elemental properties such as the nominal compositional ratio of the bulk target material [37,38,39], though this may have resulted from the loss of Ge during the deposition process at elevated temperatures because of its volatility leading to preferential inclusion of Se in the thin films fabricated at higher temperatures.

**Figure 4:**
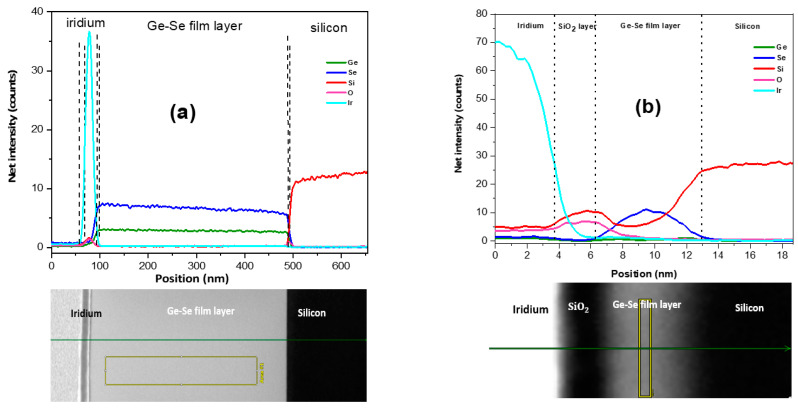
STEM images of GeSe_4_ thin film, SiO_2_, and silicon layers (bottom). EDX line scans for Ge, Se, Si, O, and Ir, were obtained along the indicated lines shown on the STEM images (**a**) 25 °C and (**b**) 600 °C.

Figure 4 illustrates STEM images and the EDX line scans acquired along the lines of the GeSe_4_ thin film cross-sections through the silicon substrate. The EDX line scan analysis of the sample fabricated at 25 °C revealed a thin film layer dominated by SiO_4_ and iridium on top of the GeSe_4_ thin film layer, as depicted in Figure 4a. The line scan profile analysis indicated that the deposited layer is composed of massive Ge and Se material without any interdiffusion. On the other hand, the sample prepared at 600 °C has a Ge-Se layer diffused into the silicon substrate, to form a homogeneous layer of Ge-Se-diffused-silicon (Ge-Se-Si), as shown in Figure 4b. However, Ge is present only in meagre quantities. Therefore, the STEM image and EDX scan profile of Figure 4b clearly show a significant diffusion of Se with O impurities into the silicon substrate.

**Figure 5:**
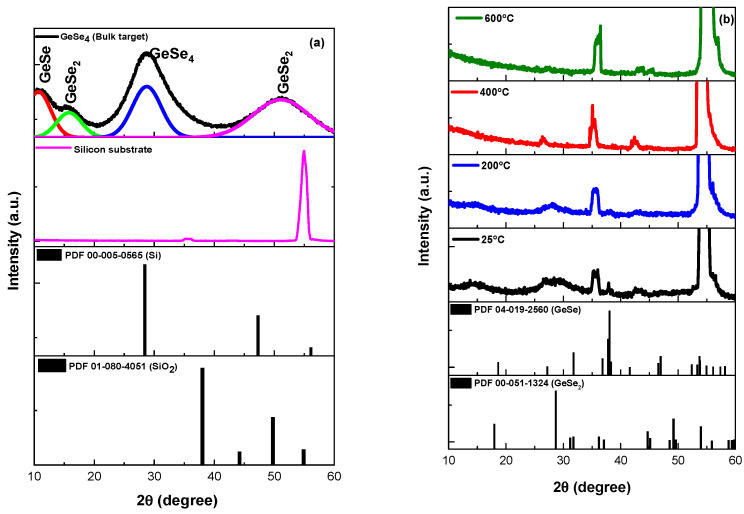
XRD patterns for (**a**) a pure silicon substrate (thickness = 675 μm) and GeSe4 bulk glass, (**b**) thin films fabricated on silicon glass substrates by fs-PLD at various substrate temperatures 25, 200, 400, and 600 °C.

Figure 5 displays the XRD patterns of the silicon substrate, GeSe4 bulk target glass, and Ge-Se thin films deposited at various substrate temperatures. The XRD patterns obtained from the silicon substrate in Figure 5a exhibit a strong diffraction peak centred at 2θ = 56.07 and indexed as (311). It corresponds to Bragg’s reflection of cubic silicon [40,41,42,43,44,45,46,47,48,49] with the International Centre for Diffraction Data (ICDD) reference pattern No. 00-005-0565. In addition, the diffraction peak at 2θ = ~36° (200) is identified as silica, which has ICDD reference pattern No. 00-080-4051. Furthermore, the XRD pattern of the bulk GeSe_4_ target glass in Figure 5a reveals four distinct wide-stretched amorphous halo structures pinpointed at 2θ = 13, 17, 28, and 51° without any sharp peaks, which are fitted with multiple Gaussian functions. These wide-stretched bands correspond to amorphous phases of GeSe, GeSe2, and GeSe4 [21,43,44,45,46]

Further characterisation was conducted using the XRD to study the influence of substrate temperature on the Ge-Se thin films deposited with fs-PLD. The more intense diffraction peak at 2θ = 56° identified in the silicon substrate is also observed in all the thin film samples. The XRD diffraction patterns of samples fabricated at a substrate temperature of 25 °C exhibit similar diffraction with the peaks centred at 2θ = 15, 29.5° and 43°, which are identical to the Ge-Se target. Furthermore, two broad amorphous structures range from 2θ = 10 to 20° and 35 to 36° and these correspond to stretched amorphous halo structures of the target. The XRD pattern of the sample deposited at 25 °C reveals additional peaks at 2θ = ~27°, 38° and 53°, indexed as the Ge-Se crystalline structure, as shown in Figure 5b. These diffraction peaks match those of Ge-Se orthorhombic phase (ICDD reference code: 04-019-2560). However, as the substrate temperature increases to 400 °C, the intensity of these broad, amorphous diffraction peaks first diminishes and then disappears completely. For instance, the GeSe_4_ amorphous structures observed from the samples deposited at 25 °C and 200 °C disappeared completely in the sample fabricated at 600 °C owing to high volatility and a decrease in the film thickness at such a high substrate temperature. The absence of the GeSe_4_ amorphous phase in samples with a substrate temperature of 400 °C and 600 °C could be attributed to the low thin film thickness (~7 nm) and the diffused form of Ge and Se in the silica/silicon substrate, which may not be sensitive enough for the XRD instrument to detect [42].

**Figure 6:**
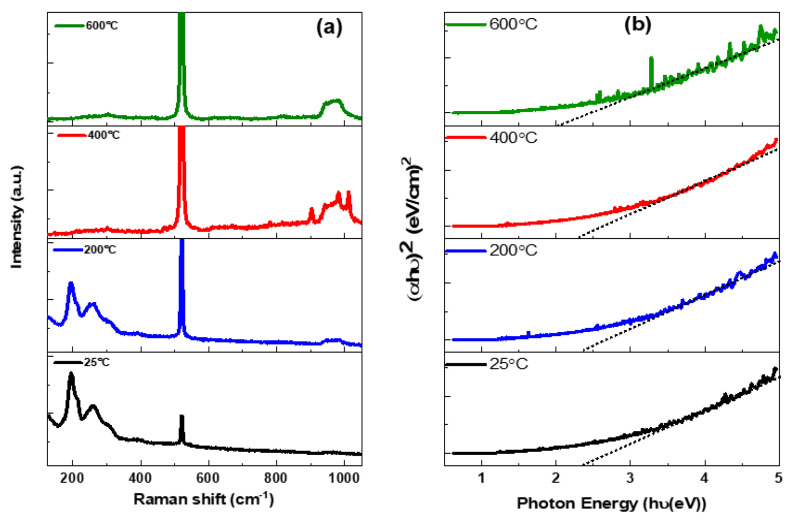
As-deposited GeSe4 thin films at various substrate temperatures:(**a**) Raman spectra, and (**b**) Tauc plots.

Figure 6a illustrates Raman spectra of as-deposited GeSe_4_ thin films at a substrate temperature range from 25 °C to 600 °C. Samples fabricated at 25 and 200 °C exhibit four prominent peaks centred at 197, 216, and 302 cm^−1^ which correspond to Ge-Se corner-sharing clusters of GeSe_4_ tetrahedra, edge-sharing clusters of GeSe_4_ tetrahedra, and asymmetrical stretching of edge-sharing bonds [47,48,49]. Nevertheless, as the substrate temperature increases from 400 °C to 600 °C, a broad stretching vibration band’s integrated intensity, ranging from 180 cm^−1^ to 230 cm^−1^, disappears entirely. The decrease in intensity of the vibration band with a peak at 302 cm^−1^ is another change observed in the Raman spectra. Thus, the changes in the Raman spectra at the substrate temperatures of 400 °C and 600 °C can be ascribed to the diffusing and volatility of Se and Ge, as discussed before, leading to complete removal of the deposited GeSe_4_ at 600 °C. The signatures of the diffused layer are hardly visible in the spectra due to its very low thickness.

In addition, optical transmission and reflectance spectra (not shown) of as-deposited thin films at various substrate temperatures were recorded by a Perkin Elmer Lambda 905 UV–VIS–NIR Spectrophotometer equipped with an integrating sphere in the wavelength range 250–2000 nm. The Lambert’s law equation was utilised to calculate the absorption coefficient expressed as in [50], as follows;

(1) $$\alpha =\frac{1}{l}\ln\left(\frac{{\left(1-R\right)}^{2}}{T}\right)$$

where *R* and *T* are the reflectance and transmission, and *l* is the thickness of the film. The optical bandgap of transition between the valence and conduction bands was obtained using the Tauc power relation [50].

(2) $$\alpha h\upsilon =A{\left(h\upsilon -E_{g}\right)}^{n}$$

where *h* is the Planck’s constant, *υ* is frequency, *E_g_* is the optical bandgap, *A* is a proportionality constant, and *n* isa probability factor that takes a value of 1/2 (direct allowed). Figure 4 illustrates optical absorption coefficient, (αhυ)^2^, against the photon energy, hυ. The optical bandgaps were determined by extrapolating a straight line onto the hυ -axis intercepts as shown in Figure 4b, which resulted in optical bandgaps of 2.4, 2.4, 2.3 and 2.0 eV from thin film samples fabricated at substrate temperatures of 25, 200, 400, and 600 °C, respectively. The decreased optical bandgap as the substrate temperature increases could be attributed to the formation of different Ge-Se phases, as already discussed in the XRD and Raman spectroscopy analysis. Therefore, the decrease in the optical bandgap of these samples fabricated by fs-PLD is ascribed to the reduction of the deposited thin film at higher temperatures.

## 4. Conclusions

In the present study, fs-PLD was employed to deposit and dope Ge-Se thin films onto a silicon (100) substrate at various temperatures. The influence of substrate temperature on the structural and optical absorption mechanism of Ge-Se thin films prepared was investigated. The substrate temperature significantly impacts the structural, cross-sectional, thickness, and optical characterisations of Ge-Se film layers deposited on the silicon substrate. The Ge-Se thin film cross-sectional analysis reveals that an increase in substrate temperature decreases film thickness and optical bandgap. This result suggests that ultrafast fs-PLD can be utilised to fabricate thicker chalcogenide glass thin films at room temperature with no defects or voids in the thin film and thereby improve optical properties, which could be beneficial for chalcogenide device applications.

## Data Availability

The data presented in this study are available on request from the corresponding author.
